# Constipation: On knife edge

**DOI:** 10.1002/jpr3.12079

**Published:** 2024-05-14

**Authors:** Michelle M. Corrado, Melissa Wong, Laura Z. Fenton, Steven Moulton, Alexandra L. Kilgore

**Affiliations:** ^1^ Section of Pediatric Gastroenterology, Hepatology, and Nutrition, Digestive Health Institute, Children's Hospital Colorado University of Colorado Aurora Colorado USA; ^2^ Department of Pediatrics University of Colorado School of Medicine Aurora Colorado USA; ^3^ Department of Radiology, Division of Pediatric‐Radiology, Children's Hospital Colorado University of Colorado Aurora Colorado USA; ^4^ Department of Surgery, Division of Pediatric Surgery University of Colorado School of Medicine Aurora Colorado USA; ^5^ Department of Pediatric Surgery Children's Hospital Colorado Aurora Colorado USA

**Keywords:** endoscopy, foreign body ingestion, surgery

A 19‐year‐old with trisomy 21, autism spectrum disorder, developmental delays, and foreign body (FB) ingestions was followed by pediatric gastroenterology for chronic retentive constipation. The family contacted gastroenterology for concern of fecal impaction. The nonverbal patient was described as “off” with poor sleep, inappetence, and eructation. The patient had no fevers or emesis. Family endorsed daily encopresis and enuresis per baseline. Given a history of declining exams and after shared‐decision making, the provider obtained an abdominal X‐ray (AXR) to evaluate stool burden.

The AXR was notable for a radiopaque FB consistent with a steak knife measuring 23 cm in length, obliquely oriented and likely within the stomach, extending into the duodenum without pneumoperitoneum (Figure 1). There was a moderate amount of stool in the colon. Endoscopic removal was considered, but thought to pose a risk of injury to the esophagus and posterior pharynx during removal of the knife. The patient was therefore admitted for an exploratory laparotomy with gastrotomy and FB removal. The patient remained inpatient for 6 days and diet was advanced after fluoroscopic imaging demonstrated no upper gastrointestinal leak. There have been no additional FB ingestions.

**Figure 1:**
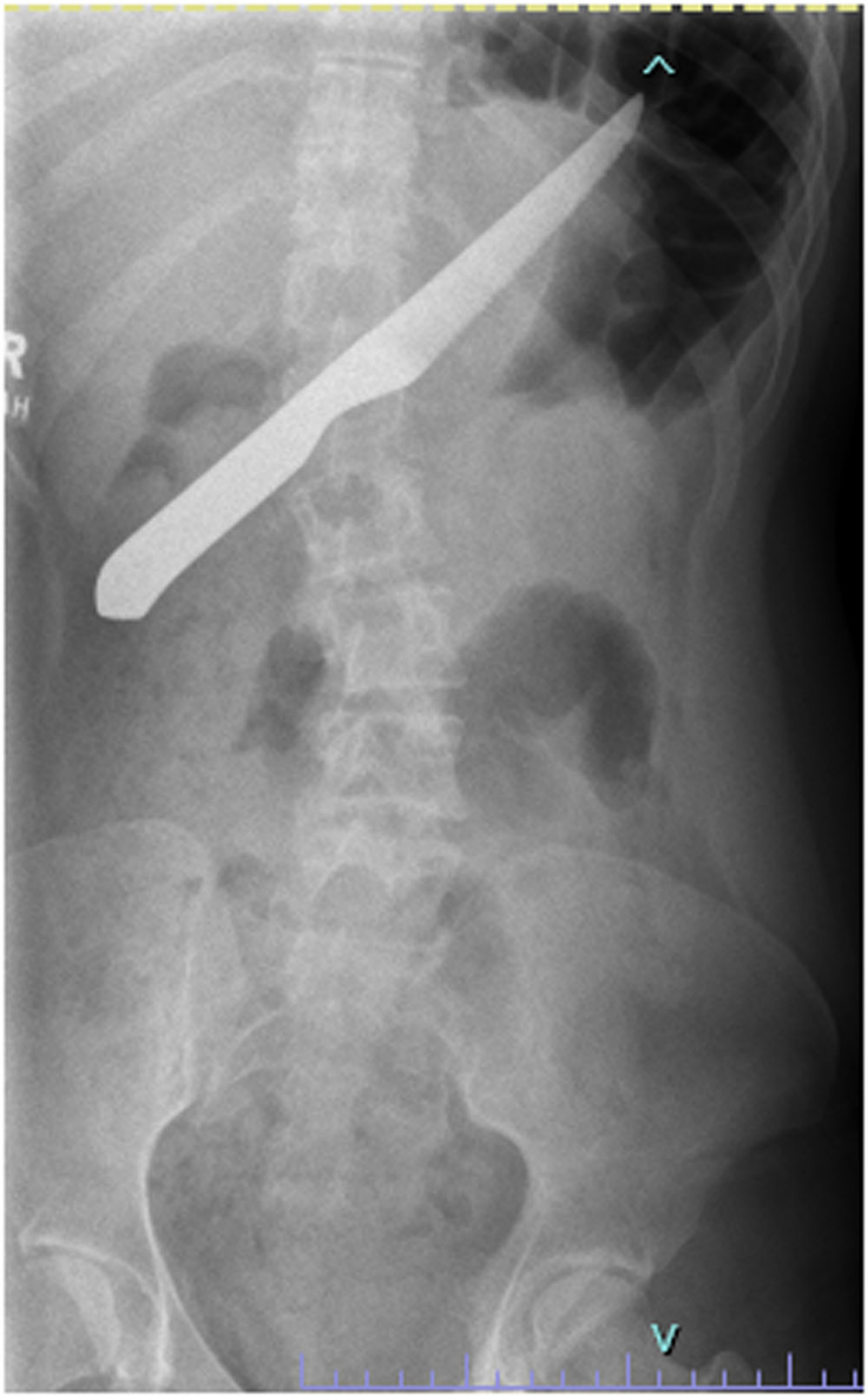
One‐view abdominal X‐ray demonstrated a 23 cm radiopaque foreign body consistent with swallowed steak knife, obliquely oriented in the upper abdomen, likely within the stomach and extending into the first portion of the duodenum. There was no pneumoperitoneum on left lateral decubitus view. Bowel gas was present and without obstruction. There was a moderate amount of stool in the colon.

It is important to consider a broad differential for gastrointestinal complaints and to specifically ask about FB ingestions, including button batteries, magnets, and chemical ingestions.^1^ Most FB spontaneously pass (90%) with few requiring surgical intervention (<1%).^2^, ^3^ The decision between endoscopic versus surgical removal should be made within a multidisciplinary team pending the FB object, location, and duration in the gastrointestinal tract.^4^

## CONFLICT OF INTEREST STATEMENT

The authors declare no conflict of interest.

## ETHICS STATEMENT

Consent by the parents of the patient has been obtained; they are aware of and agree with the intent for this article to be published.
